# The effectiveness of rapid sequence intubation (RSI) versus non-RSI in emergency department: an analysis of multicenter prospective observational study

**DOI:** 10.1186/s12245-017-0129-8

**Published:** 2017-01-25

**Authors:** Masashi Okubo, Koichiro Gibo, Yusuke Hagiwara, Yukiko Nakayama, Kohei Hasegawa, On behalf of the Japanese Emergency Medicine Network Investigators

**Affiliations:** 1 Department of Emergency Medicine, University of Pittsburgh, Iroquois Building Suite 400 A, 3600 Forbes Avenue, Pittsburgh, PA 15261 USA; 2 Biostatistics Center, Kurume University, 67 Asahimachi, Kurume, Fukuoka 830-0011 Japan; 3 Department of Pediatric Emergency and Critical Care Medicine, Tokyo Metropolitan Children’s Medical Center, 2-8-29 Musashidai, Fuchu, Tokyo 183-8561 Japan; 4 Department of Emergency Medicine, Okinawa Prefectural Chubu Hospital, 281 Miyazato, Uruma, Okinawa 904-2293 Japan; 5 Department of Emergency Medicine, Massachusetts General Hospital, 55 Fruit Street, Boston, MA 02114 USA

**Keywords:** Airway management, Intubation, Rapid sequence intubation, Intubation success, Complications of intubation, Resuscitation, Emergency department

## Abstract

**Background:**

Although rapid sequence intubation (RSI) is the method of choice in emergency department (ED) airway management, data to support the use of RSI remain scarce. We sought to compare the effectiveness of airway management between RSI and non-RSI (intubation with sedative agents only or without medications) in the ED.

**Methods:**

Secondary analysis of the data from a multicenter prospective observational registry at 13 Japanese EDs. All non-cardiac-arrest patients who underwent intubation with RSI or non-RSI were included for the analysis. Outcomes of interest were the success rate of intubation and intubation-related complications.

**Results:**

Of 2365 eligible patients, 761 (32%) underwent intubations with RSI and 1,604 (68%) with non-RSI. Intubations with RSI had a higher success rate on the first attempt compared to those with non-RSI (73 vs. 63%; *P* < 0.0001). By contrast, the complication rates did not differ significantly between RSI and non-RSI groups (12 vs. 13%; *P* = 0.59). After adjusting for age, sex, estimated weight, principal indication, device, specialties and training level of the intubator, and clustering of patients within EDs, intubation with RSI was associated with a significantly higher success rate on the first attempt (OR, 2.3; 95% CI, 1.8–2.9; *P* < 0.0001) while that with RSI was not associated with the risk of complications (OR, 0.9; 95% CI, 0.6–1.2; *P* = 0.31).

**Conclusions:**

In this large multicenter study of ED airway management, we found that intubation with RSI was independently associated with a higher success rate on the first attempt but not with the risk of complications.

## Background

Intubation is a critical procedure performed in emergency departments (EDs). Rapid sequence intubation (RSI) is the most commonly used method of ED intubation in many nations [1–8]. Previous studies have reported the associations between the use of RSI and high-intubation success rates and low complication rates [4, 7–13]. However, their inferences are potentially limited by a lack of a control group [7], small sample sizes [4, 11, 12], lack of adjustment for potential confounding factors and clustering [8], and limited generalizability (e.g., studies in the operating room settings [9, 10], in the pre-hospital setting [11], in a pediatric population [4], and conducted in a single or two centers [7, 12, 13]). Despite its clinical relevance, there have been no large multicenter studies to compare the effectiveness of RSI to non-RSI methods with adjustment for potential confounding factors and clustering in the ED setting.

To address the knowledge gap in the literature, we analyzed data from a multicenter prospective observational study to investigate intubation success and complication rates with the use of RSI compared to those of non-RSI methods in the ED.

## Methods

### Study design and setting

This study was a secondary analysis of the data from the first Japanese Emergency Airway Network (JEAN-1) Study, a multicenter prospective observational study designed to characterize the current ED airway management across Japan. The study setting, methods of measurement, and measured variables are described elsewhere [14–18]. In sum, JEAN-1 is a consortium of 13 academic and community medical centers from different geographic regions across Japan. All 13 EDs were staffed by emergency attending physicians; 12 had affiliations with emergency medicine residency training programs. Emergency attending physicians were defined as postgraduate years 6 or more; emergency medicine resident physicians were defined as postgraduate years 3, 4, or 5. Participating institutions included level I (*n* = 11) or level II equivalent (*n* = 2) trauma centers with a median ED census of 25,000 patient visits per year (range, 4200–67,000). Each ED maintained individual protocols, policies, and procedures for ED airway management. Intubations were performed by attending physicians or by resident physicians at the discretion of supervising ED attending physicians. The institutional review board of each participating center approved the protocol with waiver of informed consent prior to data collection.

### Selection of participants

All adults and children who underwent emergency intubation during a 29-month period (April 2010 to August 2012) were eligible for inclusion in analyses. As intubations without medications are the widely adopted initial method in patients with cardiac arrest, we excluded this population from the analysis [19]. We also excluded patients who underwent intubation with paralytics alone, flexible fiberoptic intubation, blind nasal intubation, or surgical cricothyrotomy to compare the outcomes between oral intubations with RSI and those with non-RSI. In addition, we excluded patients with multiple intubation attempts using an alternative method (e.g., changes in the intubation method from non-RSI to RSI) from the analysis for the secondary outcome to compare the outcomes between intubations with RSI and those with non-RSI that used the same intubation method both on the first and second attempt (i.e., we excluded patients who underwent the second intubation attempt with different methods).

### Methods of measurement

After each ED intubation, the operator completed a standardized data collection form that included the patient age, sex, primary indication for intubation, method of intubation, all medications used to facilitate intubation, device, specialties and training level of intubator, number of attempts, success or failure, and complications [14–18]. Methods of intubation, medications and dosages, and devices were chosen at the discretion of operators. We monitored compliance with data form completion by reviewing professional billing records. Where the data collection form was missing, we interviewed the involved physicians to ascertain airway management details. These *post hoc* interviews occurred within 2 weeks of the patient encounters.

### Outcome measures

The primary outcome measure was successful on the first intubation attempt. The secondary outcome measures were successful within the second attempt and intubation-associated complications (overall and major). Intubation success was defined as the proper placement of an endotracheal tube through the vocal cord confirmed by quantitative or colorimetric end-tidal CO_2_ monitoring [20]. An intubation “attempt” was defined as a single insertion of the laryngoscopy past the teeth [15]. Complications were defined as cardiac arrest, hypotension, hypoxemia, regurgitation, esophageal intubation with delayed recognition, main stem bronchial intubation, dental or lip trauma, airway trauma, or allergic reaction [17]. Cardiac arrest included asystole, pulseless electric activity, or dysrhythmia with non-measurable blood pressure [17]. Hypotension was defined as systolic blood pressure less than 90 mmHg [17]. Hypoxemia was defined as pulse oximetry saturation less than 90% during an intubation attempt, not secondary to esophageal intubation [17]. We also defined major complications as cardiac arrest, hypotension, hypoxemia, regurgitation, or esophageal intubation with delayed recognition [17].

### Statistical analysis

We first analyzed the compiled data with simple descriptive statistics. Continuous data are presented as means and standard deviations (SD); categorical data are reported as proportions. Next, we compared outcomes between RSI and non-RSI methods in ED patients who underwent intubation attempts. RSI was defined as the administration of a potent induction agent followed immediately by a rapidly acting paralytic agent to induce unconsciousness and motor paralysis for intubation [1, 8, 15, 19, 21]. Non-RSI was defined as intubation with sedative agent only or intubation without medications. We fit three unconditional logistic regression models: (1) unadjusted model, (2) adjusted model for selected variables, and (3) adjusted random-effects model for selected variables and potential clustering of patients within EDs, with each of the four outcome measures as dependent variables. Based on *a priori* knowledge, we chose a set of potential confounders (age, sex, estimated weight, principal indication for intubation, device, and the specialties and training level of intubator) [3, 7, 8, 13, 22]. In the regression model, the predictive effects on outcomes were assessed for RSI with non-RSI as a reference group.

In the sensitivity analyses, first, we repeated the multivariable analysis stratifying the non-RSI methods into intubation with sedative agent only and intubation without medications. Second, we repeated the model in a subgroup of patients at the level I trauma centers. Lastly, we repeated the model excluding intubations with slow-onset medications, such as midazolam, diazepam, haloperidol, and vecuronium from the RSI group. All odds ratios (ORs) were presented with 95% confidence intervals (CIs). All tests were 2-tailed, and *P* < 0.05 was regarded as statistically significant. Data analyses were conducted with JMP statistical software (version 10; SAS Institute, Inc., Cary, NC) and R software, version 3.0.2. (www.r-project.org).

## Results

**Fig. 1:**
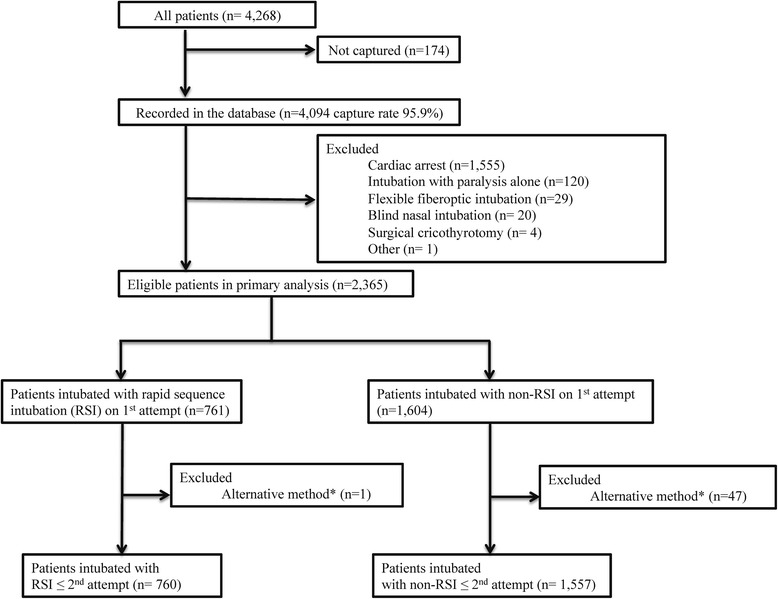
Patients receiving tracheal intubations in the emergency department. ^*^Changes in intubation method (e.g., from non-RSI to RSI)

During the 29-month period, there were 4268 patients requiring emergency airway management at the 13 EDs (Fig. 1). Among these, 4094 intubations were recorded in the database (capture rate, 96%). From this cohort, we excluded 1555 patients with cardiac arrest and 174 patients who underwent an initial intubation attempt with paralytics alone, flexible fiberoptic intubation, blind nasal intubation, cricothyrotomy, or other methods. After these exclusions, we analyzed 2365 patients for the primary outcome. Of these, RSI was used for 761 patients (32%) and non-RSI was used for 1604 patients (68%). For the secondary outcomes, we further excluded 48 patients with subsequent intubation attempts using an alternative intubation method: fiberoptic intubation (*n* = 1), RSI (*n* = 26), blind nasal intubation (*n* = 1), cricothyrotomy (*n* = 4), intubation with paralytics alone (*n* = 4), and intubation with sedative agent only (*n* = 9) at the second attempt. After these exclusions, the analytic cohort comprised 2317 ED patients for the secondary outcomes.

### Patient and airway management characteristics

The mean age of patients was 61 years, and the majority was male (Table 1). Most intubations involved medical emergencies. RSI was less commonly used for medical encounters (77%) compared to non-RSI methods (83%). By contrast, RSI was more commonly used for trauma encounters (23%) than non-RSI (17%).

**Table 1:** Characteristics of 2365 patients receiving intubation in the emergency department^a^

Patient characteristics	RSI	Non-RSI
	(*n* = 761)	(*n* = 1604)
Age, mean (SD), years	61 (20)	61 (21)
Age ≤18 years (%)	23 (3%)	64 (4%)
Age <2 years (%)	4 (0.5%)	18 (1%)
Age 2–9 years (%)	7 (0.9%)	22 (1%)
Age 10–18 years (%)	12 (2%)	24 (1%)
Male sex (%)	470 (62%)	941 (59%)
Body weight, mean (SD), kg	59 (15)	59 (17)
Primary indication (%)		
Medical encounters	589 (77%)	1333 (83%)
Respiratory failure	239 (31%)	303 (19%)
Altered mental status	231 (30%)	712 (44%)
Shock	93 (13%)	216 (13%)
Airway obstruction	11 (1%)	78 (5%)
Asthma	7 (0.9%)	10 (0.6%)
Other medical	8 (1%)	14 (0.8%)
Trauma encounters	172 (23%)	271 (17%)
Head trauma	65 (9%)	138 (9%)
Shock	55 (7%)	60 (4%)
Burn/inhalation	23 (3%)	16 (1%)
Facial/neck trauma	15 (2%)	35 (2%)
Other trauma	14 (2%)	22 (1%)

*Abbreviation*: *RSI* rapid sequence intubation, *SD* standard deviation

^a^Percentage may not equal 100 because of rounding

The most frequently used sedative agent was midazolam in the RSI group and propofol in the non-RSI group (Table 2). The most common paralytic agent used in RSI was rocuronium. Among the patients intubated with non-RSI methods, intubation with sedative agent only was used in 54% and intubation without medications in 45%. Direct laryngoscopy was used 98% in the RSI group and 96% in the non-RSI group.

**Table 2:** Airway management characteristics in 2365 patients receiving intubation in the emergency department^a^

	RSI	Non-RSI
	(*n* = 761)	(*n* = 1604)
Initial method (%)		
Rapid sequence intubation (RSI)	761 (100%)	0
Intubation with sedative agent only	0	871 (54%)
Intubation without medications	0	729 (45%)
Other^b^	0	4 (0.2%)
Sedatives (%)		
No sedatives	0	733 (46%)
Midazolam	427 (56%)	255 (16%)
Dosage, mean (SD), mg/kg	0.09 (0.07)	0.1 (0.07)
Diazepam	104 (14%)	71 (4%)
Dosage, mean (SD), mg/kg	0.2 (0.19)	0.2 (0.08)
Propofol	103 (14%)	406 (25%)
Dosage, mean (SD), mg/kg	1.3 (0.8)	1.1 (0.6)
Ketamine	87 (11%)	37 (2%)
Dosage, mean (SD), mg/kg	1.0 (0.3)	1.0 (0.6)
Combination with any of the included sedative categories	7 (0.9%)	18 (1%)
Other^c^	33 (4%)	84 (5%)
Paralytics (%)		
No paralytic	0	1604 (100%)
Rocuronium	531 (70%)	0
Dosage, mean (SD), mg/kg	0.9 (0.3)	
Vecuronium	161 (21%)	0
Dosage, mean (SD), mg/kg	0.1 (0.04)	
Succinylcholine	69 (9%)	0
Dosage, mean (SD), mg/kg	1.1 (0.4)	
Device (%)		
Direct laryngoscopy	746 (98%)	1545 (96%)
Direct laryngoscopy + gum elastic bougie	2 (0.2%)	7 (0.4%)
Video laryngoscopy	13 (2%)	50 (3%)
Video laryngoscopy + gum elastic bougie	0	1 (0.06%)
Lighted stylet	0	1 (0.06%)
Specialty of first intubator (%)		
Transitional year resident^d^	321 (42%)	462 (29%)
Emergency medicine resident	141 (19%)	679 (42%)
Emergency physician	177 (23%)	305 (19%)
Other specialty^e^	122 (16%)	156 (10%)
Unknown	0	2 (0.1%)

*Abbreviation*: *RSI* rapid sequence intubation, *SD* standard deviation

^a^Percentage may not equal 100 because of rounding

^b^Defined as oral intubation using topical anesthesia, lidocaine, or atropine

^c^Defined as administration of fentanyl, morphine, buprenorphine, pentazocine, or haloperidol

^d^Defined as post graduate years 1 or 2

^e^Defined as internal medicine, surgery, anesthesia, or pediatrics

### Intubation successes and complications

In the unadjusted analysis (Table 3), intubations with RSI method had a higher chance of success both on the first attempt (73% [95% CI, 69–76%] vs. 63% [95% CI, 60–65%]; unadjusted OR 1.6; 95% CI, 1.3–1.9; *P* < 0.0001) and within the second attempt (90% [95% CI, 88–92%] vs. 87% [95% CI, 85–88%]; unadjusted OR 1.5; 95% CI, 1.1–1.9; *P* < 0.01) compared to those with non-RSI methods. Likewise, intubation with RSI was independently associated with a higher chance of intubation success both on the first attempt (OR 2.1; 95% CI, 1.7–2.6; *P* < 0.0001) and within the second attempt (OR 1.8; 95% CI, 1.4–2.6; *P* < 0.0001) after adjusting for potential confounders (Table 4; Appendix 1: Table 5). The adjusted association persisted with the use of random-effects model accounting for clustering within the EDs, in the subgroup analysis of patients in the level-I trauma centers (Appendix 2: Table 6), with stratification by non-RSI method (Table 4), and with stratification by RSI method (Appendix 3: Table 7).

**Table 3:** Unadjusted associations of intubation method with intubation outcomes

*n* (%, 95% CI)	Success on 1st attempt	Success ≤2nd attempt	Complication^a^	Major complication^b^
RSI				
(*n* = 761 on 1st attempt, *n* = 760 ≤ 2nd attempt)	553 (73%, 69–76%)	687 (90%, 88–92%)	93 (12%, 10–15%)	62 (8%, 6–10%)
Non-RSI				
(*n* = 1604 on 1st attempt, *n* = 1557 ≤ 2nd attempt)	1003 (63%, 60–65%)	1348 (87%, 85–88%)	209 (13%, 11–15%)	122 (8%, 6–9%)
Intubation with sedative agent only				
(*n* = 871 on 1st attempt, *n* = 849 ≤ 2nd attempt)	542 (62%, 59–65%)	724 (85%, 83–88%)	121 (14%, 12–16%)	72 (8%, 7–10%)
Intubation without medications				
(*n* = 729 on 1st attempt, *n* = 706 ≤ 2nd attempt)	460 (63%, 60–66%)	622 (88%, 86 –90%)	86 (12%, 10–14%)	50 (7%, 5–9%)

*Abbreviation*: *RSI* rapid sequence intubation, *CI* confidence interval

^**a**^Defined as cardiac arrest, hypotension, hypoxemia, regurgitation, esophageal intubation with delayed recognition, main stem bronchial intubation, dental or lip trauma, airway trauma, or allergic reaction

^**b**^Defined as cardiac arrest, hypotension, hypoxemia, regurgitation, or esophageal intubation with delayed recognition

**Table 4:** Multivariable associations of intubation methods with intubation outcomes

	Success on 1st attempt, adjusted OR (95% CI) *P* value	Success ≤2nd attempt, adjusted OR (95% CI) *P* value	Complications^c^, adjusted OR (95% CI) *P* value	Major complications^d^, adjusted OR (95% CI) *P* value
RSI versus non-RSI				
Multivariable model adjusting for selected variables^a^	2.1 (1.7–2.6) *P* < 0.0001	1.8 (1.4–2.6) *P* < 0.0001	1.0 (0.7–1.3) *P* = 0.76	1.0 (0.7–1.5) *P* = 0.76
Multivariable model adjusting for selected variables and clustering of patients^b^	2.3 (1.8–2.9) *P* < 0.0001	1.9 (1.1–3.0) *P* < 0.0001	0.9 (0.6–1.2) *P* = 0.31	0.9 (0.6–1.3) *P* = 0.53
Stratified analysis by non-RSI methods adjusting for selected variables^a^				
RSI vs. intubation with sedative agent only	2.2 (1.7–2.8) *P* < 0.0001	2.2 (1.6–3.0) *P* < 0.0001	0.8 (0.6–1.2) *P* = 0.30	0.9 (0.6–1.3) *P* = 0.63
RSI vs. intubation without medications	2.0 (1.6–2.6) *P* < 0.0001	1.5 (1.0–2.1) *P* = 0.03	1.1 (0.8–1.5) *P* = 0.54	1.2 (0.8–1.9) *P* = 0.35

*Abbreviation*: *OR* odds ratio, *CI* confidence interval, *RSI* rapid sequence intubation

^a^Adjusted for age, sex, estimated body weight, principal indication for intubation, device, and the specialties and training level of intubator

^b^Adjusted for age, sex, estimated body weight, principal indication for intubation, device, the specialties and training level of intubator, and potential clustering of patients within EDs with random effects model

^**c**^Defined as cardiac arrest, hypotension, hypoxemia, regurgitation, esophageal intubation with delayed recognition, main stem bronchial intubation, dental or lip trauma, airway trauma, or allergic reaction

^**d**^Defined as cardiac arrest, hypotension, hypoxemia, regurgitation, or esophageal intubation

By contrast, between the RSI and non-RSI methods, there was no significant difference in the unadjusted complication rate (12% [95% CI, 10–15%] vs. 13% [95% CI, 11–15%]; unadjusted OR 0.9; 95% CI, 0.7–1.2; *P* = 0.58) or in the major complication rate (8% [95% CI, 6–10%] vs. 8% [95% CI, 6–9%]; unadjusted OR 1.1; 95% CI, 0.8–1.5; *P* = 0.65; Table 3; Appendix 4: Table 8). The multivariable adjusted models and stratified analysis confirmed these null findings in the complication rates (Table 4; Appendix 1: Table 5).

## Discussion

In this large prospective study of 2365 ED patients who underwent intubation attempts in the ED, we found that intubation attempt using RSI was associated with a higher chance of success both on the first attempt and within second attempt, compared to that using non-RSI. These significant associations persisted across various statistical assumptions. By contrast, we also found no significant differences in the risk of intubation-related complication between the two intubation methods.

Several previous studies have reported a high-success intubation rate and low complication rate with the use of RSI [4, 7–13]. For example, in the descriptive analysis of a prospective observational study (the National Emergency Airway Registry (NEAR)) of airway management in North American EDs [8], Walls et al. reported that RSI was the most frequently used initial method chosen (69%) and that the unadjusted success rate of RSI on the first attempt was higher than that of intubations with sedation without paralytics (82 vs. 76%). They also found that the unadjusted complication rate in patients intubated with RSI was lower than that with sedation without paralytics (11 vs. 16%). Similarly, in a prospective observational study of 1478 intubations at two academic EDs in Korea, Kim et al. reported that RSI was associated with a higher success rate on the first attempt [13]. Our data corroborate these findings and expand these prior researches by using more robust statistical approach in a different patient population and practice setting. Along with the existing literature, our findings lend significant support to the current ED management—RSI as the method of choice.

There are several plausible mechanisms of the observed higher success with the use of RSI than with non-RSI methods. The literature in the operating room setting demonstrated that intubations with RSI lead to better intubating conditions such as abducted vocal cords, lack of vocal cord movement, ease of laryngoscopy, and lack of cough reflex when compared to those with sedatives only [9, 10]. Alternatively, patient selections by indication—e.g., patients who underwent intubation with RSI were less likely to have had predicted difficult laryngoscopy—may have contributed to the higher success rate with the use of RSI. Indeed, the guidelines and experts recommend that the RSI methods should be avoided for patients with predicted difficult laryngoscopy [23].

We found no significant difference in the complication rate between the RSI and non-RSI groups, although the previous studies reported that intubations with RSI had a lower rate of complications [8, 12]. The reasons of this discrepancies are likely multifactorial—e.g., differences in study design, setting, data measurement, definition of outcomes, or any combination of these factors. Alternatively, it is possible that, in our study population, non-RSI methods were used more frequently and potentially less selectively (i.e., non-RSI methods were also used for patients with less-difficult airway), which may have resulted in a relatively lower rate of complications in the non-RSI group, compared with previous studies, thereby leading to the null results [5, 7, 8].

We are struck with the discrepancies not only in the use of RSI but also in the success rates between our study and previous studies. For example, Walls et al. reported the first-pass success rates using RSI of 82% and intubation with sedation only of 76% [8] while the rates in our study were 73 and 63%, respectively. The reasons of those discrepancies are likely multifactorial, such as the wide inter-hospital variation in emergency airway management, lack of standard education of emergency airway management, lack of accreditation of emergency medicine training program in Japan, or any combination of these factors [15, 24].

### Potential limitations

Our study has several potential limitations. First, surveillance systems used in this study are subject to self-reporting bias, thereby leading overestimation of success rate and underestimation of complication rates. However, these non-differential misclassifications may not have biased our inferences as over and under estimations could evenly occur in the both groups. Second, this registry was not designed to assess patient outcomes after ED disposition such as in-hospital mortality. Third, the dosage of rocuronium, the most commonly used paralytics in this registry, was lower (0.9 mg/kg) than the standard dosage in RSI (1.0 mg/kg) [19]. However, the finding that intubation using RSI even with suboptimal dosage of paralytics demonstrated a higher success rate than that using non-RSI supports the favorable effectiveness of RSI. Fourth, some factors of airway management (e.g., preoxygenation, cricoid pressure, predicted airway difficulty, peri-intubation vital signs, rationale of medication dosage, and time-related factors such as duration between medication administration to intubation) were not assessed in this registry. Fifth, as with any observational study, the association between RSI method and a higher chance of intubation success does not necessarily prove causality and may be confounded by unmeasured factors, such as patient’s underlying comorbidity, airway difficulty, and differences in procedural skill. High-quality clinical trials of ED airway management would be instrumental in demonstrating causality between airway management methods and outcomes. However, such trials are ethically difficult given the existing literature showing a superiority of the RSI compared to the non-RSI techniques in the ED setting. Additionally, it is well documented that patients who consent to participate in the controlled framework of a clinical trial may be systematically different from the general population [25]. As an alternative, our large prospective multicenter data in the “real world” setting reflect the effectiveness of ED airway management, therefore enhancing the potential generalizability of the findings.

## Conclusions

In conclusion, in this large multicenter prospective observational study in Japanese EDs, intubations with RSI had a higher chance of intubation success both on the first and within second attempt but no significant difference in the risk of complication when compared to the non-RSI methods. For clinicians, these findings lend significant support to the use of RSI in the ED by providing more robust evidence of its superior effectiveness in emergency airway management. For researchers, because the evidence for accurately predicting patients in whom RSI should be avoided remains to be elucidated, our data should facilitate further investigation on risk stratification of patients who require airway management in the ED.

## Appendix 1

**Table 5:** Multivariable model of rapid sequence intubation versus non-rapid sequence intubation

	Success on 1st attempt	Success ≤2nd attempt, adjusted OR (95% CI)	Complication^a^, adjusted OR (95% CI)	Major complication^b^, adjusted OR (95% CI)
Primary exposure				
RSI (vs. non-RSI)	2.1 (1.7–2.6)	1.8 (1.4–2.6)	1.0 (0.7–1.3)	1.0 (0.7–1.5)
Covariates				
Age decile	1.0 (1.0–1.0)	1.0 (1.0–1.0)	1.0 (1.0–1.0)	1.0 (1.0–1.0)
Male (vs. female)	0.8 (0.7–1.0)	0.8 (0.6–1.1)	0.8 (0.6–1.0)	0.9 (0.6–1.3)
Estimated body weight decile	1.0 (1.0–1.0)	1.0 (1.0–1.0)	1.0 (1.0–1.0)	1.0 (1.0–1.0)
Primary indication				
Medical indication^c^	1 [reference]	1 [reference]	1 [reference]	1 [reference]
Trauma^d^	0.6 (0.5–0.7)	0.7 (0.5–1.0)	1.1 (0.7–1.3)	0.9 (0.6–1.5)
Device				
Direct laryngoscopy	1 [reference]	1 [reference]	1 [reference]	1 [reference]
Video laryngoscopy	0.6 (0.3–1.0)	0.4 (0.2–0.8)	1.5 (0.7–2.7)	0.9 (0.3–2.2)
Other^e^	1.0 (0.3–3.7)	0.2 (0.1–0.5)	1.4 (0.5–3.5)	0.9 (0.1–3.2)
Intubator				
Emergency physician	1 [reference]	1 [reference]	1 [reference]	1 [reference]
Transitional-year resident^f^	0.2 (0.2–0.3)	0.2 (0.1–0.3)	1.2 (0.9–1.8)	1.0 (0.6–1.6)
Emergency medicine resident	0.7 (0.5–0.9)	0.8 (0.5–1.1)	1.1 (0.8–1.5)	0.6 (0.4–1.0)
Other^g^	0.4 (0.3–0.5)	0.4 (0.3–0.7)	1.3 (0.9–2.1)	1.8 (1.1–3.0)

*Abbreviation*: *OR* odds ratio, *CI* confidential interval, *RSI* rapid sequence intubation

^a^Defined as cardiac arrest, hypotension, hypoxemia, regurgitation, esophageal intubation with delayed recognition, main stem bronchial intubation, dental or lip trauma, airway trauma, or allergic reaction

^b^Defined as cardiac arrest, hypotension, hypoxemia, regurgitation, or esophageal intubation

^c^Defined as altered mental status, respiratory failure, shock, airway obstruction, asthma, or other medical indications

^d^Defined as head trauma, shock, facial/neck trauma, burn/inhalation, or other trauma indications

^e^Defined as direct laryngoscopy or video laryngoscopy with gum elastic bougie or lighted stylet

^f^Defined as postgraduate years 1 or 2

^g^Defined as surgery, anesthesia, or pediatrics

## Appendix 2

**Table 6:** Random-effects model of rapid sequence intubation versus non-rapid sequence intubation in all patients and patients at level I trauma centers

	Success on 1st attempt (all patients [*n* = 2365]), Adjusted OR (95% CI)	Success on 1st attempt (patients at level I trauma centers [*n* = 2147]), Adjusted OR (95% CI)
Primary exposure		
RSI (vs. non-RSI)	2.3 (1.8–2.9)	2.0 (1.6–2.6)
Covariates		
Age decile	1.0 (1.0–1.0)	1.0 (1.0–1.0)
Male (vs. female)	0.8 (0.7–1.0)	0.8 (0.6–0.9)
Estimated body weight decile	1.0 (1.0–1.0)	1.0 (1.0–1.0)
Primary indication		
Medical indication^a^	1 [reference]	1 [reference]
Trauma^b^	0.6 (0.5–0.8)	0.6 (0.5–0.8)
Device		
Direct laryngoscopy	1 [reference]	1 [reference]
Video laryngoscopy	0.6 (0.4–1.1)	0.7 (0.3–1.6)
Other^c^	0.9 (0.3–3.4)	0.9 (0.2–3.3)
Intubator		
Emergency physician	1 [reference]	1 [reference]
Transitional-year resident^d^	0.2 (0.1–0.3)	0.2 (0.1–0.3)
Emergency medicine resident	0.5 (0.4–0.7)	0.6 (0.4–0.8)
Other^e^	0.3 (0.2–0.5)	0.4 (0.3–0.6)

^a^Defined as altered mental status, respiratory failure, shock, airway obstruction, asthma, or other medical indications

^b^Defined as head trauma, shock, facial/neck trauma, burn/inhalation, or other trauma indications

^c^Defined as direct laryngoscopy or video laryngoscopy with gum elastic bougie or lighted stylet

^d^Defined as postgraduate years 1 or 2

^e^Defined as surgery, anesthesia, or pediatrics

## Appendix 3

**Table 7:** Multivariable association of stratified RSI with intubation outcomes^a^

	Success on 1st attempt, Adjusted OR (95% CI) *P* value	Success ≤2nd attempt, Adjusted OR (95% CI) *P* value	Complications^c^, Adjusted OR (95% CI) *P* value	Major complications^d^, Adjusted OR (95% CI) *P* value
Modified RSI^b^ vs. non-RSI	2.2 (1.5–3.2) *P* = 0.0012	1.4 (0.9–2.4) *P* = 0.15	0.7 (0.4–1.2) *P* = 0.19	0.6 (0.3–1.2) *P* = 0.16
Modified RSI^b^ vs. intubation with sedative agent only	2.4 (1.6–3.6) *P* < 0.0001	1.7 (1.0–2.9) *P* = 0.05	0.6 (0.4–1.1) *P* = 0.11	0.5 (0.2–1.0) *P* = 0.07
Modified RSI^b^ vs. intubation without medications	1.9 (1.3–3.0) *P* = 0.0006	1.1 (0.7–2.0) *P* = 0.64	0.9 (0.5–1.5) *P* = 0.55	0.8 (0.3–1.6) *P* = 0.53

*Abbreviation*: *OR* odds ratio, *CI* confidence interval, *RSI* rapid sequence intubation

^a^Adjusted for age, sex, estimated body weight, principal indication for intubation, device, and the specialties and training level of intubator

^b^Excluded cases that used vecuronium, midazolam, diazepam, and haloperidol

^c^Defined as cardiac arrest, hypotension, hypoxemia, regurgitation, esophageal intubation with delayed recognition, main stem bronchial intubation, dental or lip trauma, airway trauma, or allergic reaction

^d^Defined as cardiac arrest, hypotension, hypoxemia, regurgitation, or esophageal intubation with delayed recognition

## Appendix 4

**Table 8:** Complications in 2317 patients receiving intubation in the emergency department^a^

Complications (%)	RSI (*n* = 760)	Non-RSI (*n* = 1557)	Intubation with sedative agent only (*n* = 849)	Intubation without medications (*n* = 706)
Cardiac arrest	5 (<1)	3 (<1)	1 (<1)	2 (<1)
Hypotension	26 (3)	25 (2)	24 (3)	1 (<1)
Hypoxemia	1 (<1)	5 (<1)	2 (<1)	3 (<1)
Regurgitation	8 (1)	30 (2)	14 (2)	16 (2)
Esophageal intubation with delayed recognition	27 (4)	74 (5)	40 (5)	34 (5)
Main stem bronchial intubation	14 (2)	20 (1)	7 (<1)	13 (2)
Dental or lip trauma	13 (2)	70 (4)	43 (5)	27 (4)
Airway trauma	3 (<1)	5 (<1)	1 (<1)	4 (<1)
Allergic reaction	1 (<1)	0 (0)	0 (0)	0 (0)

^a^Patients may have more than one complication

## Abbreviations

CI: Confidence interval
ED: Emergency department
JEAN: Japanese emergency airway network
NEAR: National emergency airway registry
OR: Odds ratio
RSI: Rapid sequence intubation
SD: Standard deviation
